# CLEAR: A Holistic Figure-of-Merit for Post- and Predicting Electronic and Photonic-based Compute-system Evolution

**DOI:** 10.1038/s41598-020-63408-7

**Published:** 2020-04-16

**Authors:** Shuai Sun, Vikram K. Narayana, Mario Miscuglio, Lionel C. Kimerling, Tarek El-Ghazawi, Volker J. Sorger

**Affiliations:** 1 Department of Electrical and Computer Engineering, George Washington University, 800 22nd Science & Engineering Hall, Washington, DC 20052 USA; 2 Department of Materials Science and Engineering, Massachusetts Institute of Technology, 77 Massachusetts Avenue Cambridge, Cambridge, MA 02139 USA

**Keywords:** Electrical and electronic engineering, Information technology

## Abstract

Continuing demands for increased computing efficiency and communication bandwidth have pushed the current semiconductor technology to its limit. This led to novel technologies with the potential to outperform conventional electronic solutions such as photonic pre-processors or accelerators, electronic-photonic hybrid circuits, and neural networks. However, the efforts made to describe and predict the performance evolution of compute-performance fall short to accurately predict and thereby explain the actually observed development pace with time; that is all proposed metrics eventually deviate from their development trajectory after several years from when they were originally proposed. This discrepancy demands a figure-of-merit that includes a holistic set of driving forces of the compute-system evolution. Here we introduce the Capability-to-Latency-Energy-Amount-Resistance (CLEAR) metric encompassing synchronizing speed, energy efficiency, physical machine size scaling, and economic cost. We show that CLEAR is the only metric to accurately describe the historical compute-system development. We find that even across different technology options CLEAR matches the observed (post-diction) constant rate-of-growth, and also fits proposed future compute-system (prediction). Therefore, we propose CLEAR to serve as a guide to quantitatively predict required compute-system demands at a given time in the future.

## Introduction

**Figure 1:**
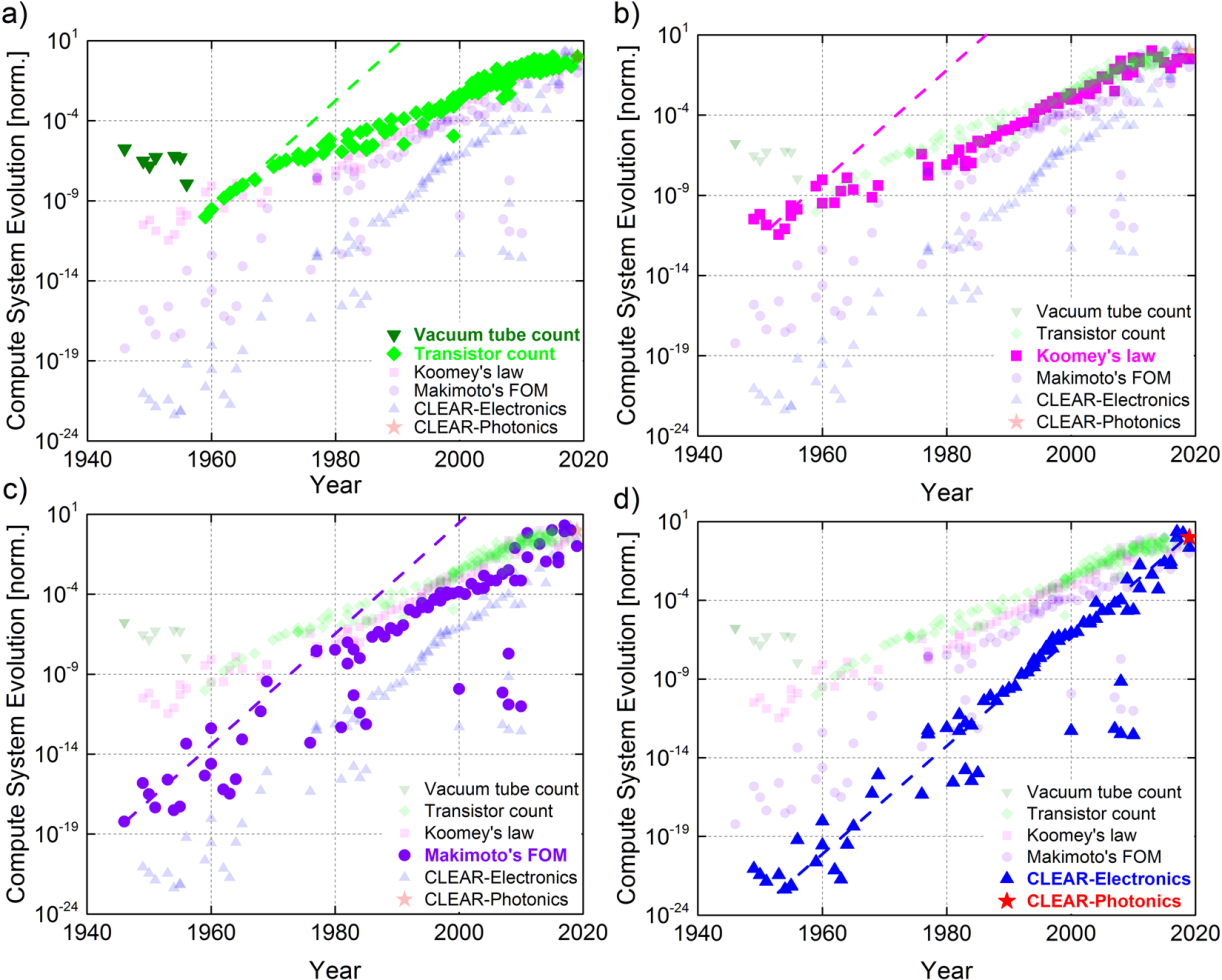
The Computing system evolution trends starting from the year 1946 to the present day based on four different figure-of-merit: (**a**) Moore’s law which counts the number of the components on-chip in unit of transistor numbers; (**b**) Koomey’s law which represents the energy efficiency per computing in unit of bit/Joule; (**c**) Makimoto’s figure-of-merit that includes the 'intelligence', power, size and the cost of the system in unit of MIPS/(W·mm^3^·$); and (**d**) CLEAR (defined in Eq. (4)) that also considers the latency of a system in addition to Makimoto’s figure-of-merit as well as the economic resistance to a new-technology adoption units: MIPS/(sec·W·mm^3^·$). Moreover, the Photonic CLEAR data are plotted based on Intel’s forecast for silicon photonics. Dashed lines represent the linear fit (in log-scale) based on the initial growth rate, with an annual performance doubling rate. Transparency has been adjusted in each figure for better visibility.

While the evolution of computing performance is ever monotonically increasing, the observed device scaling pace of the semiconductor industry, however, is notably slowing down especially since the 14 nm technology node transistors^1,2^. This is driven by both physical limitations and the economic resistance of the non-circumventable fabrication process control. As a result, Moore’s law, as the guiding map of the semiconductor industry, has been revised multiple times to compromise these obstacles^3^. Similarly, the original development slope (vs. time) of other metrics, such as the Koomey’s law which describes the computing energy efficiency and the Makimoto’s figure-of-merit (FOM) which covers the compute power and the related energy, size and cost, both deviate compared to the observed technology development pace (Fig. 1) after a certain driving force (e.g. multi-core, fabrication cost) is unable to provide exponential scaling improvements. For instance, the processor performance of *N*-core systems is still limited by $\frac{1}{(1-p)+\,{}^{p}/{}_{N}}$ where *p* represents the parallelisation portion^4^. Therefore, tracing the evolution of compute performance is becoming more challenging by using Moore’s Law (or other prediction metrics) alone, which only use a single or a few driving factors to describe the compute-system’s performance^3,5^. Furthermore, different hardware implementations’ (e.g. electrical, optical) performance advantages scale differently with time, thus further challenge making evolution predictions. For example, integrated photonics, and possibly plasmonics, could augment certain communication pathways on board, or even on-chip to mitigate power and heat dissipation challenges, and extend data bandwidth with the potential of breaking through the electronic digital efficiency wall by utilizing concepts such as wavelength division multiplexing (WDM), optical angular momentum, or higher modulation formats such as polarization amplitude modulation (e.g. QAM) where both phase and amplitude polarization is used concurrently^6,7^. Regarding other technology tradeoffs, a single electronic transistor with 14 nm technology node has over 3 orders of magnitude smaller footprint than a photonics microdisk ring modulator, however, photonics offers link-level interconnectivity with no capacitive charging/discharging wires, while being synergistic to the aforementioned unique characteristicswhich support up to Tbps level data rates^8^.

Here, we introduce a holistic figure-of-merit (FOM) termed Capability-to-Latency-Energy-Amount-Resistance (CLEAR) and show that its underlying driving forces accurately track historic compute performance data. As such it is the only known FOM to date to do so, to our knowledge. This FOM covers both physical and economic factors related to the evolution rate of different compute technology options. Comparing CLEAR with other FOMs that use fewer descriptive factors, such as Moore’s law, the Koomey’s law, and the Makimoto’s FOM, this 5-factor CLEAR provides the most accurate and seamless post-diction of the historical compute performance evolution since the 1940s of different technologies. As such CLEAR can be used as a technology-agnostic quantitative guideline for emerging technology options since it incorporates both fundamental physical and economic models, thus far. Based on the observations and our analysis, we draw two conclusions; (i) the compute-system evolution is monotonically increasing at a constant rate, whilst other FOMs deviate from their original tracking speed; (ii) integrated photonics (or any emerging technologies in general) can only substitute the current dominating technology if its overall performance (i.e. CLEAR value) lies at (or above) this constant evolution trend line. The remainder of this paper is structured into three major parts as follows: (1) we derive the holistic FOM CLEAR and test it using historical data to track compute-system performance and compare it with other FOMs from the semiconductor industry; (2) we perform a breakdown analysis answering which CLEAR-factors support or fail as development driving forces; and finally (3) use CLEAR to consider technology substitution prediction for electronics and photonics technologies. Note, details and technology models are provided in the Supplementary File^9^.

## Compute-system Evolution

### Dominant driving forces

All fundamental physics, semiconductor process control, and economic pressure demand ongoing changes and adaptations for technology development of compute-system^1^. Since the foundation of the semiconductor industry, Moore’s law is the governing law of has changed its underlying driving-force factors more than once before; from counting transistors the industry pivoted (1^st^ transition) to transistor footprint- and cost-scaling due to the limitations of on-chip size and complexity^5^. A second transition occurred when the clock frequency became limited due to the power density dissipation constraints described by the Dennard Scaling^10^. With transistor scaling nearing fundamental physical limits, the transistor count continues to increase, for now, driven by the parallelism introduced with multi-core and massively parallel heterogeneous architectures. This, however, worsens the communication bottleneck, while leading to the necessity to turn-off certain areas of the chip (i.e. ‘dark silicon’)^11^. As such, the device growth rate adjusted from the initial doubling every 12-month down to about 24-month today.

Recently another driving force from a different domain has emerged to affect the evolution of compute-systems, which is on-chip photonics and (or) hybridized nanophotonics, where light signal routing is performed by passive integrated photonic components while electro-optic active components can include emerging solutions such as (i) novel reconfigurable materials, (ii) strong light-matter interactions such as in plasmonics or epsilion-near-zero (ENZ) photonics, together delivering high data channel and link capacity outperforming conventional electronics at both intra-chip and inter-chip levels^12,13^. For these emerging technologies, simply counting the number of components on-chip, or the scaling the footprint and cost, as a stand-alone metric is unfeasible, as it no longer accurately reflects the actual performance evolution; this is in part due to a trend of other emerging technologies such as used in optical communication, which allows to package multiple signals with different wavelengths into the same physical channel and, thus, improves hardware utilization. Such a unique characteristic of, in this case, the optical domain, is a significant factor in system performance evaluations. Therefore, a holistic FOM that contains a plurality of driving forces is of demand to enable accurate comparisons amongst varying compute technologies in this ‘golden time of hardware development^14^.

### The holistic figure-of-merit for compute-systems

In order to derive such a technology-agnostic FOM for a compute-system, we collected performance data from PCs, laptops, mobile devices, and supercomputers since the 1940’s up until recently and here evaluate the time-evolution of the various FOMs discussed above (Fig. 1). Specifically, Moore’s law counts the number of transistors as a 1-factor FOM (Eq. 1), whilst Koomey’s law calculates the computation energy efficiency in the unit of bits per second (bps) per joule as a 2-factor FOM (Eq. 2). The million-instructions-per-second (MIPS) per unit size-cost-power are known as Makimoto’s FOM as a 4-factor FOM (Eq. 3)^15–17^.

1 $${\rm{Moore}}\mbox{'}{\rm{s}}\,{\rm{Law}}={Number}\,{of}\,{Transistors}\,[{\rm{a}}{\rm{.u.}}]$$

2 $${\rm{Koomey}}\mbox{'}{\rm{s}}\,{\rm{Law}}=\frac{Computations}{Energy}\,[{\rm{bps}}/{\rm{joule}}]$$

3 $${\rm{Makimot}}\mbox{'}{\rm{s}}\,{\rm{FOM}}=\frac{Intelligence}{Size\times Cost\times Power}\,[{{\rm{MIPS}}/({\rm{mm}}}^{3}\cdot \$\cdot {\rm{W}})]$$

The values of these three FOMs reveal a similar growth pattern; the rising tendency tracks their initial data well, but only for a limited period of time, and eventually deviate from it. This suggests that factors considered in previous FOMs were unable to fully capture the actual driving force that dominates the historical pace of compute-system evolution. Analyzing these trend lines shows that the transistor count initially (1950s-1960s) tracks the 2×/year growth rate well (dashed light green line, Fig. 1). However, the energy efficiency scaling (i.e. Koomey's law) became a dominant factor during the next period (1960s-1970s) since simply adding more transistors is limited by the size and the complexity of the chip. Therefore, Moore’s law started to deviate from the 2×/year trend while Makimoto’s FOM still maintained its original growth rate. Starting from the late 1970s, both the size and power scaling slowly become saturated due to the fabrication yield, energy leakage, and heat dissipation challenges. Together with the emergence of parallelism (i.e. multi-core processors) and the economic scaling in the market, Makimoto’s FOM finally deviates as well (starting around 1978). The dashed lines shown in Fig. 1 represent the initial performance improvement predictions by each law, (e.g. Moore’s Law a doubling of chip-components count every year). These trend lines show how each additional factor introduced, by the respective laws, impacts its own original prediction metric shown as a deviation from this original trend. Treating this trend as a theoretical technology evolution rate upper limit, now one can understand whether the ‘claimed’ trend is indeed the ‘actual’ one or not; that is, whether the law-at-hand is the slow-down reason with technology and time-development, or whether new performance factors start to become dominant. Thus, as of today, there is no clear known FOM that can (a) explain the latest performance developments, and (b) provides a guideline by predicting future performance. The introduced holistic CLEAR FOM encompasses performance factors from a multitude of technology options that incorporates both physical and economic constraints. However, our main claim is that the factors making up CLEAR are not randomly selected, but are rather fundamental to computing, and to economic forces. CLEAR is given by

4 $${\rm{C}}{\rm{L}}{\rm{E}}{\rm{A}}{\rm{R}}=\frac{Capability}{Latency\times Energy\times Amount\times Resistence}\,[{\rm{M}}{\rm{I}}{\rm{P}}{\rm{S}}/(s\,\cdot \,{\rm{W}}\,\cdot \,{{\rm{m}}{\rm{m}}}^{3}\,\cdot {\rm{\$}})]$$

Termed Capability-to-Latency-Energy-Amount-Resistance (CLEAR, Eq. 4), this FOM is able to accurately track (without deviation) the compute-system evolution for the first time (Fig. 1d). That is, we find a constant growth rate through the entire compute-evolution spanning 4-orders of magnitude performance improvements over seven decades. Moreover, the actual observed evolution rate is consistently held at 2× for every 12-months. This 5-factor FOM is defined based on the performance vs. cost concept, and can be applied at a device, circuit, and system level. For instance, at the system level, CLEAR breaks down as follows: the capability (C) is the system performance given by the product of million-instructions-per-second (MIPS) and the instruction length; the minimum latency (L) relates to the clock frequency and is limited by the temporal window between two adjacent clock cycles; ‘energy’ (E) represents the rate of energy consumption for operating such system to obtain certain Capacity expressed in units of Watt; the amount (A) represents the spatial volume (i.e. physical size) of the system and is a function of the process dimensionality; the resistance (R) quantifies the economic resistance against a new technology adoption of the market. It is an economic model based on the Boston Consulting Group (BCG) experience curve, which explains the relation between the cumulative production and the unit cost^18^. We derive the linear relation between the log scale of unit price and time and confirm this relation by fitting historical data from ref. ^19,20^ with the CLEAR FOM. We note that the metric MIPS as a measure of performance is being replaced by metrics such as floating-point operations (FLOPS) due to its susceptibility to the underlying instruction set. We apply CLEAR to the various historical processors for which other performance metrics are not available under known benchmarking suites (for example SPEC or LINPAC). Towards making MIPS a representative performance metric, however, we weighted (i.e. multiplied) each instruction by its length, thus giving the relative general metric in units of bit-per-second. A detailed description of all the FOM models used in Fig. 1 is presented in the Supplementary File^9^.

### Compute-system evolution trend discussion

In order to determine the time period of the FOM accurately tracking machine development of (i.e. applicability), we take the deviation point where the data deviates away from a single exponential growth rate. After comparing all four FOMs shown in Fig. 1, we find that when a FOM incorporates more relevant factors its starting point of deviation from its original trend occurs later. Unlike CLEAR, however, which shows an accurate fit to the data throughout. Thus, we empirically find, that computing performance grows consistently at a fixed rate at about 2×/year, and is independent of technology. Testing emerging compute engine performance such as those augmented with photonics as predicted by IBM for example^21^, we find that such technologies indeed could continue the evolutionary 2×/year development trend (red stars in Fig. 1).

Furthermore we find that the relative deviation from the 2×/year trend line can be used to classify compute-system FOMs; for instance, the additional overhead on (i.e. physical size, parallelism, cooling, low economy-of-scale, and manufacturing costs) of supercomputers show their inferior CLEAR values relative to all other computer types such as laptops and mobile devices, despite their higher performance (dashed circles, Fig. 1c,d). The high parallelism of multi-core technologies used in supercomputers is challenged by computing-to-energy returns described by Amdahl’s law^4^. We observe that while supercomputers deliver petaflop performance, the entire infrastructure resembles that of computers 5–30 years back, thus questioning future scale-up.

## Breakdown Analysis of CLEAR

**Figure 2:**
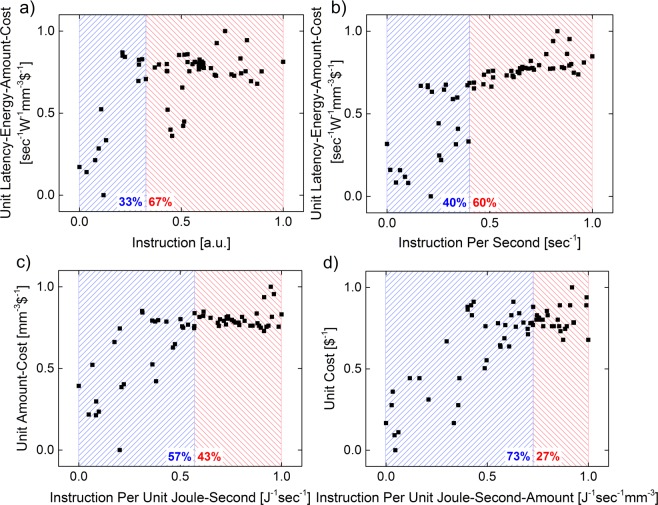
Normalized Capability to Latency-Energy-Amount-Resistant breakdown for driving force analysis. The entire C-L-E-A-R is broken-down into four groups each contains two parts: the scaling-factor which shows the factors considered along the technology evolution path (x-axis) and the revealing-factor which shows the tracking ability of the chosen factor or factor combinations (y-axis). Results show that a later deviation from the normalized development tracking is observed when more factors are included to describe computer performance. (**a**) Speed excluding capability (C’) against latency-energy-amount-cost (LEAR). (**b**) Capability (C) against latency-energy-amount-cost (LEAR). (**c**) Capability per latency-energy (CLE) against amount-cost (AR). (**d**) Capability per latency-energy-amount (CLEA) against cost (R). Both x and y-axes are normalized to unity for better comparison. The linear growth and the saturating regions are covered with blue and red shadows respectively.

To gain further insights in the relative impacts of each of the 5 factors of CLEAR, we decomposite it down into its different factors by plotting one against the remaining other to reveal the actual driving forces with time. This can be insightful for future technology options, as it reveals insights into technology bottlenecks vs. possible opportunities. The factor combinations used here in Fig. 2 are C vs. L-E-A-R, C-L-E vs. A-R, and C-L-E-A vs. R (Fig. 2, C = capability, L = latency, E = energy, A = amount, R = resistance). In addition, we show a speed-exclusive capability vs. all other factors, since the only scaling factor during the starting years of the semiconductor industry is the number of components on-chip. Moreover, it is important to mention that the relative positions of each data point are more important than the exact values in both x- and y-axes, and thus both axes are normalized to unity enabling comparison between panels. The non-normalized data from Fig. 2 can be found in the Supplementary File^9^. As the blue- and the red-shaded areas represent the linear growth and the saturating regions respectively, we clearly find consistent turning point shifts to the right with increasing numbers of considered FOM-factors for the x-axis (i.e. relative time). To understand this, the factors on the x-axis can be regarded as the driving force covered in the FOMs while values on the y-axis denote the actual tracking conditions of the driving forces being considered. Therefore, the linear region denotes that the factors on the x-axis are still dominating the technology evolution, while the driving force of the technology starts to shift to other factors when entering the saturating region. This result matches with the observation made, that the compute-system evolution is always growing with that constant speed and the divergence from the FOM happens only when other driving forces emerge.

## CLEAR in Applications

Above we show that CLEAR is an appropriate FOM to trace the information processing capability of a multitude of compute-systems with time. More importantly, the ability to seamlessly track the evolution among different technologies enables CLEAR to predict future technology substitution and to define a standard for future technology including technology hybridizations, such as between electronics and photonics circuits^22–26^. In this section, we discuss evolution predictions for the traditional electronics and hybrid photonic-plasmonics, and a device-level comparison among electronics, photonics, plasmonics, and hybrid plasmonics. An elaborate description of all the parameters, formulas and assumptions could be found in the Supplementary File^9^.

### Technology substitution

On-chip photonic interconnects have recently shown high-data capacity outperforming conventional electrical interconnects at the intra-chip level when hybridized with active plasmonic devices^12^. While optical data-routing is perceived as a possible solution to address the communication bottleneck of computer cores and is commonly deployed in data centers and supercomputers, integrated photonics has yet to be introduced into consumer electronics. This appears to be surprising at first since previous studies suggested a superior performance for photonic-plasmonic hybridization with a break-even signal propagation distance of ten’s of micrometers relative to electronics^12^. Thus, the question arises as to why integrated optics has not been used in intra-chip interconnections in mass-market products to-date?

**Figure 3:**
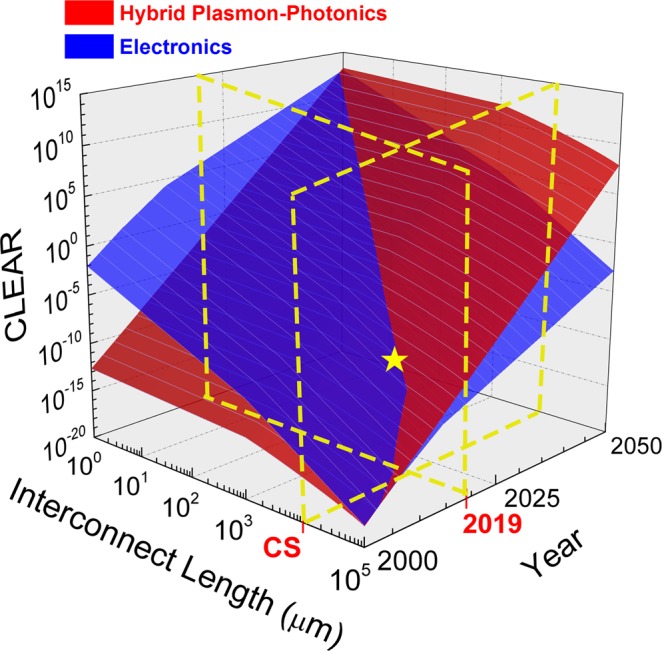
CLEAR comparison between electrical (blue) and hybrid photon-plasmon (red) on-chip interconnect links as a function of link length and technology evolution time. The chip scale (CS = 1 cm) link length and current year (2019) are denoted in red. The following models are deployed; (**a**) A capacity-area model based on the number of transistors and on-chip optical devices, which can be regarded as the original Moore’s Law model; (**b**) An energy efficiency model is derived based on Koomey’s law, which is bounded by the *k*_*B*_*T·ln(2)* ≈ 2.75 zJ/bit, Landauer limit (k_B_ is the Boltzmann constant; T is the temperature); (**c**) A the economic resistance model based on technology-experience models and at the year 2019, the electronic link cost less than one billionth to one millionth of the cost of the hybrid link; and (**d**) A model for parallelism (after year 2006) capturing multi-core architecture and the limitation from ‘dark’ silicon concepts in electrical link interconnects. The yellow data point represents crossover point between two technologies in the year of 2019, where the Hybrid Plasmon-Photonics technology just passed the chip scale and starts to show better CLEAR performance for on-chip applications.

Towards answering this question, we compare CLEAR for electronic links against hybrid photon-plasmonic links as a function of time evolution and signal propagation distance (Fig. 3). Here light manipulation is handled using plasmonic ‘active’ building blocks (source, modulator, detector, switch)^6,25,26^, whilst light propagation is handled by loss-loss photonics such as silicon or silicon-nitride platforms. Here we compare electronics against such a plasmon-photonic hybrid option because the separation of active vs. passive functionality in hybrid plasmon-photonics leads to higher performance (i.e. lower delay, higher bandwidth, lower energy-per-bit function)^6^. The resulting surface curves show that CLEAR of electronics and plasmon-photonics exhibit a break-even line (surfaces crossing, Fig. 3) that scales with both time and signal propagation distance. Interestingly, at the moment in time when this manuscript was written (2019), electronics still outperforms photonics at chip scale (CS = 1 cm) information traveling lengths. This interestingly matches the observation that electronics is still being commercially used in network-on-chips to-date and not photonics. The investments and developments in electronics over the last half-century, have thus set technology resistance (barrier-to-entry) for other technologies. Such learning-curve scaling has resulted in a transistor only costs one-billionth of a photonic device price or less^27^.

As technology and manufacturing processes improve, the performance-per-cost (i.e. CLEAR) break-even distance to communicate a bit of information shortens, due to a flatter cost curve of electronics compared to photonics, the latter following a power law with time. Moreover, the cost starts to increase further with electrical interconnects density scaling associated with added overhead due to fundamental physical challenges at sub-10 nm transistor nodes^27^. In contrast, hybrid optical links, while currently being costly, are yet to benefit from economy-of-scale fabrication processing, which is an aim of the American Institute for Manufacturing Integrated Photonics (AIM Photonics) consortium. Also, such scaling is now possible since the recent advances in nanophotonics; the concept of enhancing light-matter-interaction allow for wavelength-compact optoelectronic devices with the benefit of high energy efficiency and fast operating speed due to the low electrical capacitance^7^. As a result, the break-even signal propagation distance between electronics and hybrid photon-plasmonic links is expected to further shift to shorter distances with development time.  For example, the CMOS-based silicon photonic chip demonstrated by IBM in 2015 is close to the break-even area of these two technology options^28^. Indeed, integrated photonics could only be considered to substitute for electronics for communication and possible for (pre)processing as well, if its CLEAR performance growth rate could catch up with the general evolutionary trend of the compute-system.

### Computing unit evolution

In addition to applying CLEAR to the system-level for technology evolution prediction, it can be amended to guide the development of the device- and network-level alike, provided minor modifications are applied. For the device-level, for example, the signal distance becomes the device length and hence cancels in Eq. (4). Thus the area reduces to the device length. The data capacity and latency from the link become the device operating speed and response time, respectively. Thus, at the device-level, CLEAR becomes Capability-to-Length-Energy-Area-Resistance, which breaks down as follows: (i) the device operating frequency is the capability (C); (ii) the scaling efficiency which is the reciprocal of the critical scaling length (L) of the device describes the interaction length to provide functionality; (iii) the energy consumption (E) of the energy ‘cost’ per bit is the reciprocal of the energy efficiency; (iv) the on-chip footprint, or area (A), and v) the economic resistance (R) in units of dollars ($) is the reciprocal of the device cost efficiency. Here the critical scaling length in the denominator does not conflict with the area factor but indicates the scaling level or ability of the device to deliver functionality given its length. For instance, the critical scaling length of the CMOS transistor is the length of its logic gate, which controls the ON/OFF states. For photonic and plasmonic devices, it can be regarded as the laser or modulator (linear) length, or microring diameter when, for instance, ring or disc resonators are utilized.

**Figure 4:**
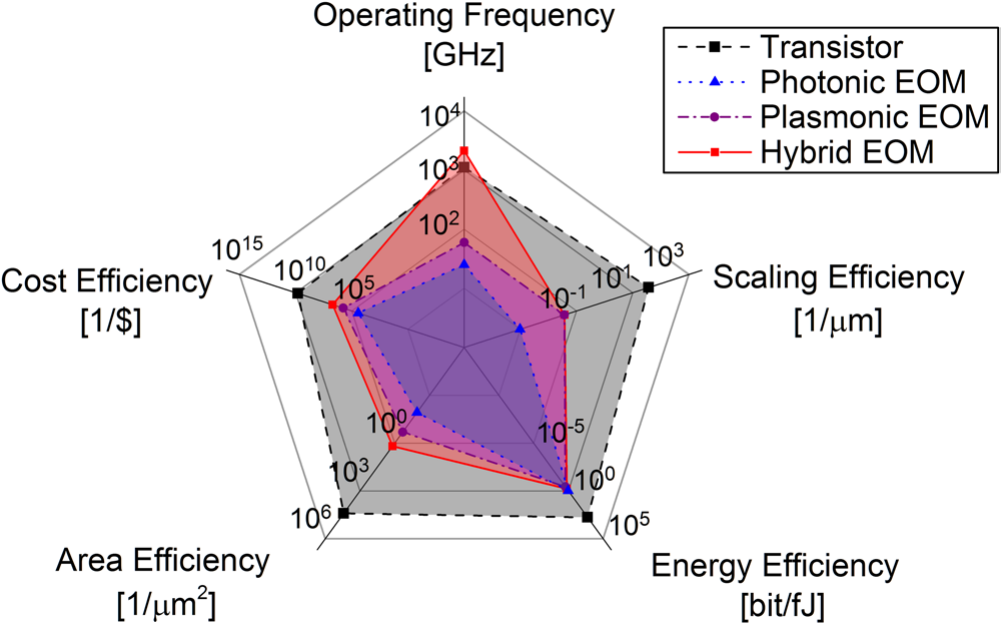
CLEAR performance break-down comparison at device level (higher value shows better performance). Each axis of the radar plot represents one factor of the device-CLEAR and is scaled to the fundamental physical limit of each factor^9^. Four devices compared from different technology options are: (1) conventional CMOS transistor at 14 nm process; (2) photonic microdisk silicon modulator^29^; (3) MOS field effect plasmonic modulator^30^; and (4) photonic plasmonic hybrid ITO modulator^31^. The colored area of each device also demonstrates the relative CLEAR value of each device.

Next, we demonstrate how to compare the performance of (a) an electronic transistor; (b) a microdisk ring modulator; (c) a plasmonic electro-optic modulator and (d) a hybrid plasmon polariton modulator among different technologies based on CLEAR FOM^29–31^. We represent the device-CLEAR results as five merit factors in a radar plot (Fig. 4). Note, each factor is shown in such a way that the larger the colored area is, the higher the CLEAR FOM of the device technology. Moreover, some of the factors of the device-CLEAR have physical constraints that fundamentally limit further growth independent of chosen technology. For example, the energy efficiency of the device is ultimately limited by the Landauer’s principle (*E*_min_ ≥ *k*_*B*_*T* ln(2), which restricts the minimum energy consumption to erase a bit of information to 2.87 zeptojoule at room temperature (T = 300 K). Given this device energy limit, the Margolus–Levitin theorem sets an upper limit for the maximum operating frequency of the device. Based on the fundamental limit of quantum computing, a device with the amount of *E* energy requires at least a time in units of *h/4E* to transfer a bit from one state to the other resulting in a switching bandwidth of about 16 THz for energy levels at the Landauer’s limit^32^. When approaching the quantum limit for data communication, the device’s critical length would be scaled down to the dimension of about 1.5 nm based on the Heisenberg Uncertainty Principle (*x*_*min*_ ≥ *ħ/*Δ*p* = *ħ*/$\sqrt{2m{E}_{{\min }}}$ = *ħ*/$\sqrt{2m{k}_{B}T{\rm{ln2}}}=1.5\,nm$). Again, these are fundamental physical considerations, and other operational constrains, such as bit-error-rate for link-level for example, will reduce the design window further.

With all five CLEAR factors in each axis in Fig. 4 normalized to their physical limits respectively, we compare the device performance of four different technologies. The results show, that the overall performance a single transistor is rather high benefitting from small *RC* delay (*R* = resistance, *C* = Capacitance) times, compact sizes due to the nanometer-small wave function of electrons, and cost-efficiency from economic learning curves over 5-decades. However, emerging technologies, such as photonics and plasmonics, are gradually catching up with the electronic devices, and their hybrid combination may exceed the transistor at operating frequencies (which are limited by the device *RC* delay of the complex circuit) due to the sub-diffraction limited active region approaching 100’s of nm small dimensions and low insertion losses due to modal silicon-plasmonics hybridization^7^. However, the burgeoning photonics-based technologies are still suffering from the yet-to-be-perfected fabrication process and incomplete economic industry system leading to high cost, low scaling- and low area efficiency. However, Silicon photonics foundries have recently accelerated their development cycle^33^. Lastly, area-wise, those emerging technologies are unable to surpass the energy efficiency of a single transistor, yet in the end the performance of emerging technologies is not only driven by component density, but also by parallelism strategies and homomorphism (i.e. synergistic matching of the to-be-processed algorithm and the underlying executing hardware)^34–36^.

## Discussion

As we have seen from the above disussions, CLEAR can be regarded as being a universal techno-economic metric not only due to its broad hierarchical applicability (device, interconnect, system levels) but also because of its ability to be tailored to a specific application such as for on-chip network hybridization^21,23,24^. In other words, CLEAR could not only be used as a figure of merit for predicting technology platform evolution, but could also compare the overall ability of technology platform(s) under different application circumstances by adding weighting exponents to each factor in Eq. (4). In this originally proposed CLEAR metric, all five factors linearly impact the CLEAR value, however, for a specific application that relies critically on a particular factor (or on a combination of factors), each factor in CLEAR could be weighted differently. To ensure comparability, even amongst such ‘customized’ metrics, one might want to enforce that the sum of all coefficient equals to 5, similar to the normalization to unity such as for the wavefunction-integral in quantum mechanics. For instance, a portable device system may have strict energy (E) and spatial volume (A) constrains, resulting in a CLEAR metric of C^0.8^L^0.8^E^1.2^A^1.2^R for such a technology. Indeed, it would be interesting to compare evaluation trends and trade-offs from different customized metrics towards allowing technology predictions into the future.

Furthermore, we can perceive future links or networks to be dynamically reconfigured enabling the chip, or subsections thereof, to change its ideal operating point to shift depending on the current application, load, battery state, etc. Such dynamically-data-driven-applications of systems (DDDAS) are indeed sought after systems because of their combine cognitive information processing capability. Indeed adapting computer systems to a multitude of constraints is expected to have synergistic overlaps with emerging information theory systems such as neuromorphic and reservoir computing, where adaptation and tuning of ‘weights’ enable machine learning, residue number system arithmetic or even on-chip Silicon Photonic optical computing^37–40^.

It is also worth to mention that, as it happened to all previous figure of merit for predicting technology platform performance, CLEAR may eventually start to deviate from its original trend as well when more unique features of physics will be adopted in emerging technology. For the time being, the factors in CLEAR adequately cover all dominate performance factors in current and near- to-mid future technologies, thus allowing also for some-level of evolution predictions at the time of writing this manuscript.

As such CLEAR may not only be a tool for road-mapping efforts and outlook forecasting but could set a path towards a hardware-enabled ‘smart’ and cognitive computer control platforms, where performance-cost tradeoffs are reassessed and optimized in real time. Thus, CLEAR can be regarded as the new Moore’s Law that holistically captures technology development trends of a variety of hierarchical application levels.

## Conclusion

We introduce a novel Figure-of-Merit (FOM), CLEAR, incorporating a holistic set of device and compute-system performance parameters including synchronizing speed, energy efficiency, volume scaling, and economic cost. This FOM is universal since it covers both physical and economic factors known to-date that contribute to the evolution of compute-systems. As such it is applicable to a variety of technologies to include electrical and optical options. Our results show that applying CLEAR to computer development over the last 70 years reveals a constant growth rate of all known compute-systems even across different technology cycles and can, therefore, be used for post- and predictions in technology development. Our detailed factor-by-factor analysis shows that other metrics aiming to describe and guide compute performance such as Moore’s law, or Koomey’s law fall short to accurately describe compute performance development. Having established that CLEAR is the only metric to accurately post-dicts historic compute performance, we show how CLEAR can be used to benchmark emerging compute technologies. Exercising CLEAR per example with integrated photonics vs. electronics, we reveal that the break-even point between these two technologies still favors electronics to date (2019) for chip-scale length scales. However, we further show predictions by IBM for integrated photonics match ‘CLEAR-demanded’ future compute performance. Indeed, similar to information theory and algorithmic photonics anticipating novel compute engines such as those based on neuromorphic computing, reservoir computing, or residue-number-system arithmetic, it is interesting and development time-saving to ask, whether those developments may be able to deliver the performance expected by the CLEAR growth trend. Finally, founded on fundamental physical principles, this novel and holistic metric has thus the potential to become the next law for the semiconducting industry for both data processing and computing.

## Supplementary information

Supplementary file.

Supplementary Dataset.
