# Supplementary material for: CLEAR: A Holistic Figure-of-Merit for Post- and Predicting Electronic and Photonic-based Compute-system Evolution
Source: Sci Rep. 2020 Apr 16;10:6482. doi: 10.1038/s41598-020-63408-7 (PMC7162934; doi:10.1038/s41598-020-63408-7)
Supplement: Supplementary file 1 — Supplementary file. [file 41598_2020_63408_MOESM1_ESM.pdf]

CLEAR: A Holistic Figure-of-Merit for Post- and Predicting Electronic and Photonic-based Compute-system Evolution

Shuai Sun,[1] Vikram K. Narayana,[1] Mario Miscuglio,[1] Lionel C. Kimerling,[2] Tarek El-Ghazawi,[1] Volker J. Sorger[1,*]

[1]*Department of Electrical and Computer Engineering, George Washington University*
800 22nd Science & Engineering Hall, Washington, DC 20052, USA
[2]*Department of Materials Science and Engineering, Massachusetts Institute of Technology*
77 Massachusetts Avenue Cambridge, Boston, MA 02139, USA
**sorger@gwu.edu*

Outline:

1. Compute System Model Analysis
1.1 System level CLEAR breakdown
1.2 Other Dominating FOMs in Compute Systems
1.3 Historical Compute System data
2. Compute System Evolution Model
3. Technology Substitution Model Analysis
3.1 Link level CLEAR model
3.2 Device Parameters for Link Comparison
3.3 Link Parameters
3.4 Fundamental physical limits of CLEAR factors
4. Compute Unit Model Analysis
4.1 Device level CLEAR model
4.2 Device Parameters
4.3 Fundamental physical limits of CLEAR factors
5. References

---

1. Compute System Model Analysis

1.1 System level CLEAR breakdown

The model of our system-level Figure of Merit (FOM) consists of Capability-to-Latency-Energy-Amount-Resistance (CLEAR), and consists of the following details:

1) **Capability:** is the product of million instructions per second (MIPS, in [million instruction/second]) times the instruction length (in [bit/instruction]). Thus, it represents the data-handling performance of a compute system. Although floating point operations per second (FLOPS) is also commonly used, MIPS is better suited in a performance comparison that includes historical systems of the 20th century with modern systems. Since the instruction length varies among different computer systems, we use the product of MIPS times the instruction length in units of bit-per-second as the general capability of any compute system to process data.
2) **Latency:** Clock speed in [second]. While clock-less or asynchronous clocking are explored, here we focus our discussions around regularly clocking systems only. Clock speed is one basic metric to compare the operating speed of different computer systems, since it presents

the minimum time-delay any bit of information is able to traverse (time of flight) inside the system.

3) **Energy:** Energy consumption of the compute system in [watt].
4) **Amount:** Volume of the system in [mm^3]. Here the volume used for each compute system includes the associated accessories and cooling infrastructure. This is critical to obtain an accurate volumetric 'cost' when comparing different types of compute systems. For example, while supercomputers deploy a large number of cores to achieve high performance, they require enormous amounts of power. In fact, modern datacenters do not have a better (Performance/Cost)-ratio than modern personal laptops. On the other side, portable computers like smartphones sacrifice the performance for size and energy efficiency. Therefore, the Amount should not simply be limited to the areal footprint, but include the volume of the entire compute system.
5) **Resistance:** The resistance represents the economic model based on the Boston Consulting Group (BCG) experience curve model in [$], which is defined as "each time the cumulative production doubles, the unit cost falls by a constant percentage" [Ref. 30 in the manuscript]. This model reveals the relation between learning curve effects and the economic phenomenon (which relates to the labor efficiency, shared experience effect, use-cost reduction, etc.) and is valid among a broad range of industries. Based on this, we derive a *log*(unit price) vs. time relationship and verified it by using the historical learning curve of a semiconductor device [R1]. Note, this relation is confirmed by the historical data of transistor cost, which shows the linear relationship between time and the logarithmic price. Using this model, we obtain a cost prediction of the silicon photonic chips and devices in the future derive based on their recent fabrication cost. Although the unit transistor cost started to rise in the recent few years and deviated from its original BCG model, we believe that the BCG model for silicon photonics will still be valid for a few decades, since we are still at the very beginning of this novel technology and there are still plenty of room left until we reach the flat bottom of its learning curve.

1.2 Other Dominating FOMs in Compute Systems

Here we provide a brief introduction and summary of the mathematical framework of our approach resulting in Figure 1 from the main manuscript, i.e. for the conventional system level FOMs. Note, some of the conventional FOMs were introduced later than 1955, but we could still reproduce those FOMs based on the historical compute system data.

Moore's law: It is an observation that the number of device elements doubles every 12-month and it was first introduced by Gordon Moore in 1965. With the development of the semiconductor industry, the device element was later modified to transistor count and the rate reduction down to 24-month. This observation is a simplistic, empirical observation that the industry uses as a roadmap of the semiconductor evolution speed. The following equation is used to reproduce the Moore's law curve in Fig. 1:

$$Moore's\ law = Number\ of\ transistors$$

Koomey's law: This is an energy efficiency metric, which assumes the doubling time of the computation per Joule to be approximately 1.57 years. However, since it is ambiguous to define the computation among different computers, we use the number of bits as a general quantifier for the various compute systems. Thus, the Koomey's law curve we show in Figure 1 has the units of [bit/Joule] based on the following equation:

$$Koomey's\ law = \frac{Capability}{Power} = \frac{MIPS \times Instruction\ Length}{Power} = \frac{bit/s}{Joule/s} = bit/Joule$$

Makimoto's FOM: This is a metric which is related to CLEAR, however, the difference between the two is that it only uses intelligence, size, cost and power but does not consider latency to describe the compute systems. This shortcoming is however critical because the latency is also a significant factor that relates to the performance and energy trade-offs. Moreover, the data bandwidth, MIPS (millions instructions per second), is a subjective metric that may vary from computer system to system. The Makimoto's FOM is given by:

$$Makimoto's\ FOM = \frac{Intelligence}{Size \times Cost \times Power} = \frac{MIPS}{Size \times Cost \times Power}$$
$$= MIPS/(m^3 \cdot \$ \cdot W)$$

1.3 Historical Compute System Data

All the data (performance, cost, size, power and other CLEAR related parameters) of the compute systems from the 1940s to 2010s are collected online from papers, computer manuals and webpages. A Microsoft Excel sheet (file name: compute system data.xlsx) of all the data could be found in the attachment together with this supplementary file.

2. Compute System Evolution Model

To proof the main assumption in the manuscript that the driving force of the compute system changes as the time goes on, it is very important to find the main driving force for each period of time and try to compare it with the history of the compute system evolution. Moreover, as it has been proven that CLEAR is the only FOM that is able to track the evolution with a linear (in log-log scale) growth rate, all the dominated driving forces could be considered covered in CLEAR. Thus, a factor breakdown analysis is the key to find the driving force for each time.

For this factor breakdown model, the goal is to first separate one single factor from CLEAR to see when the evolution rate starts to deviate from its original speed, and then add the next factor to it. Following the history of the compute system, the linear (in log-log scale) growth region is expected to be wider and wider until it becomes fully linear when all five factors (i.e. CLEAR) are considered.

3. Technology Substitution Model Analysis

To consider the actual technology substitution and make predictions in the compute systems is extremely complicated. However, recent studies on comparing data computing and data

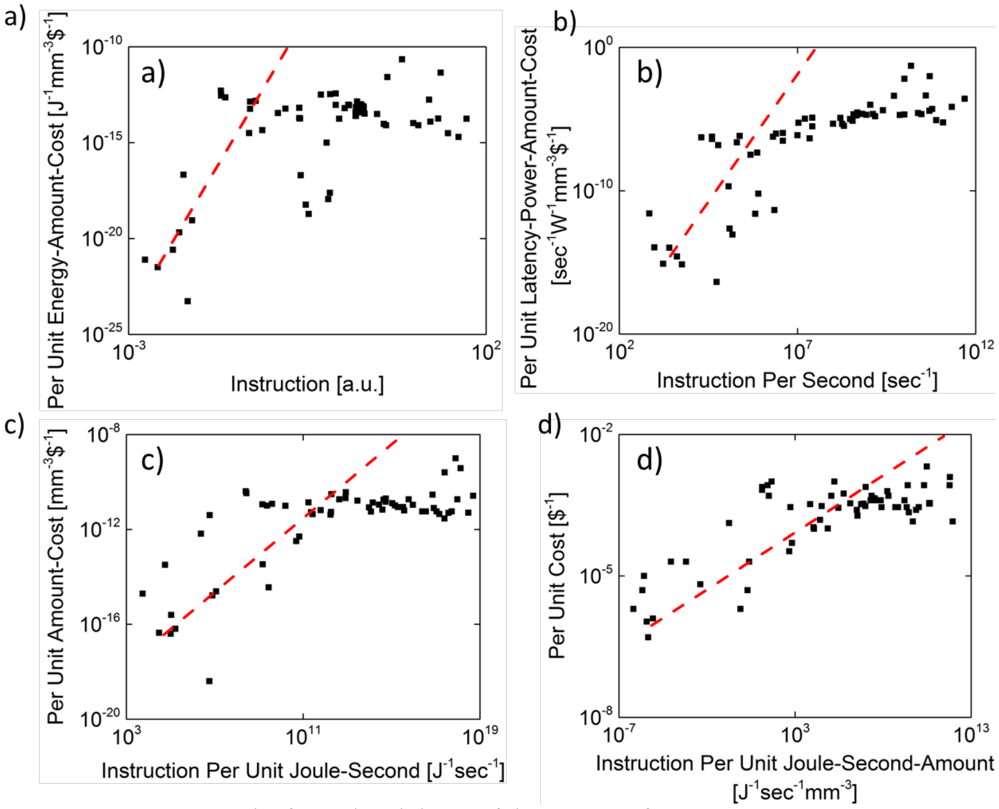

Figure S1. The factor breakdown of the CLEAR for compute systems.

communication have been done and draw the conclusion that the communication scaling is orders of magnitude more efficient and meaningful than the logic scaling since the logic building blocks are already approaching the fundamental physical limits in the quantum level [R2]. Therefore, we assume that interconnects in the system is going to eventually dominate the overall performance and all the prediction models are made for interconnects only.

3.1 Link level CLEAR model

The five-component link-level CLEAR FOM is comprised as follows:

1) **Capability:** The capability of a link in the unit of Gbps is calculated based on the Shannon equation for a noisy channel, which relates to the bandwidth of the entire channel and the signal to noise ratio.
2) **Latency:** The latency of a link is the point-to-point latency in the unit of picoseconds, which is given by the time of flight from the light source to the photodetector, and is a function of the individual waveguide's and device' model group index.
3) **Energy:** Energy consumption of a link in units of femtojoule per bit. It includes the energy consumed by the sum of active devices, and passive data routing components.
4) **Amount:** The area of a link in the unit of um^2 includes the sum of all device areas, to include the light source, waveguides, modulators, detectors, splitter, rings etc. It further incorporates

required spacing to prevent the crosstalk between adjacent waveguides based on our previous work [R3].

5) **Resistance:** For the link level, we use the economic resistance model from BCG discussed above. The optical link fabrication cost based on the total cost of an optical wafer (~$50,000) and the number of links that can fabricate on a single chip (~70,000 mm[2]).

3.2 Device Parameters for Link Comparison

All the device parameters are borrowed from our previous work [R3]. The numbers are relisted in Table S1-S3. Note, only the hybrid link (i.e. HyPPI) is showed in the comparison with electronic links in the manuscript. The reason is that for a compute system, both short (μm ~ mm) and long (mm ~ cm) distance communication is needed. For a photonic interconnect which favors long-haul data transmissions with low propagation losses, the footprint on-chip makes it unfeasible for small scales of connects due to the diffraction limits and weak light-matter interaction. However, with ultra-fast operating frequency and sub-wavelength device scale, plasmonic links become the perfect option for small distance communication, but its high ohmic loss prohibits it from longer scaling. None of these two interconnect options is able to surpass the electronic link in the full communication range (μm ~ cm) and thus we decided to only show the comparison between electronics link and hybrid plasmon-photonic link.

Table S1. Device latency (ps) for different interconnect options with link length L in μm. Shaded data are used in Fig. 2 of the manuscript.

Component	Photonic Interconnects	Plasmonic Interconnects	HyPPI
Laser/LED	N/A	5	N/A
Modulator	40	16.7	0.5
Modulator Driver	5.9	5.5	0.4
Waveguide	$8.3\times10^{-3}L$	$4.9\times10^{-3}L$	$8.3\times10^{-3}L$
Detector	25	1.4	1.4
Total (propagation + manipulation)	$8.3\times10^{-3}L+70.9$	$(4.9\times10^{-3}L+28.6)/100\mu m$	$8.3\times10^{-3}L+2.3$

Table S2. Energy efficiency (fJ/bit) of each considered device for different interconnects with link length L in μm, the minimum output current level I_{min} is 50 μA.

Component	Photonic Interconnects	Plasmonic Interconnects	HyPPI
Laser/LED	$2.5\times10^{(7.02+0.00005L)/10}$	$8.3\times10^{(8.1+0.044L)/10}$	$2.5\times10^{(8.1+0.00005L)/10}$
Source Driver	N/A	N/A	N/A
Modulator	2.77	6.80	4.25
Modulator Driver	11.88	11.08	0.74
Detector	0	0.14	0.14
Total	$14.65 + 2.5\times10^{(7.02+0.00005L)/10}$	$18.02 + 8.3\times10^{(8.1+0.044L)/10}$, $L\leq100$ μm	$5.13 + 2.5\times10^{(8.1+0.00005L)/10}$

Table S3. Power loss breaks down and detector responsivities. Link length L in the unit of μm. Shaded data are used in Fig. 2 of the manuscript.

Component	Photonic Interconnects	Plasmonic Interconnects	HyPPI-Extrinsic
**Laser Efficiency** (%)	25	20	20
**Modulator** (dB)	1.02	1.1	0.6
**Waveguide** (dB)	$5\times10^{-5}L$	$4.4\times10^{-2}L$	$5\times10^{-5}L$
**Coupler** (dB)	-	(included in the modulator part)	0.5
**Responsivity** (A/W)	0.8	0.1	0.1

Equations used in Table S1-S3 are:

$$Latency = Light\ Source\ Delay + Modulator\ Delay + Modulator\ Driver\ Delay + Detector\ Delay + Light\ Propagation\ Time$$

$$Laser\ Power = \frac{I_{min}}{BW \times Responsivity \times Efficiency} \times 10^{\frac{|loss|}{10}}$$

3.3 Link Parameters

All the optical links include three major components: light source, modulator and detector (Figure S2). The light first generated from the light source and then transmitted to the modulator which controlled by an electronic driver. After the light has been modulated, it propagates through the next segment of the waveguide and been detected by the detector and converted into the electrical domain. The devices and waveguide for each technology option may vary, but the fundamental principles are similar as we shown in Figure S2.

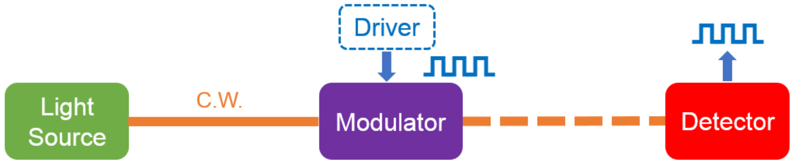

Figure S2. Schematic plot of a hybrid plasmon-photonic link.

Table S4. Link level CLEAR data. Shaded data are used in Fig. 2 of the manuscript.

100 um link length				
	**Electrical link**	**Photonic link**	**Plasmonic link**	**Hybrid link**
**Capacity (Gbps)**	4.09E+01	5.57E+02	9.76E+02	3.25E+03
**1/P2P latency (THz)**	2.51E-03	1.39E-02	1.73E-02	3.19E-01
**1/Energy (1/(fJ/bit))**	8.16E-02	3.67E-02	6.04E-03	4.70E-02
**1/Area (1/um^2)**	2.27E-01	2.78E-03	3.57E-02	2.56E-02
**1/Cost (1/$)**	1.6E+06	3.9E+03	5.0E+04	3.6E+04
**1 mm link length**				
	**Electrical link**	**Photonic link**	**Plasmonic link**	**Hybrid link**
**Capacity (Gbps)**	4.09E+01	5.57E+02	9.76E+02	3.25E+03
**1/P2P latency (ps)**	6.55E-05	1.26E-02	3.13E-03	9.41E-02
**1/Energy (1/(fJ/bit))**	1.16E-02	3.65E-02	7.43E-07	4.66E-02
**1/Area (1/um^2)**	2.27E-02	1.48E-03	4.81E-03	2.82E-03
**1/Unit Cost (1/$)**	1.6E+05	2.1E+03	6.7E+03	4.0E+03
**1 cm link length**				
	**Electrical link**	**Photonic link**	**Plasmonic link**	**Hybrid link**
**Capacity (Gbps)**	4.09E+01	5.57E+02	9.76E+02	3.25E+03
**1/P2P latency (ps)**	7.80E-07	6.49E-03	3.40E-04	1.17E-02
**1/Energy (1/fJ)**	1.21E-03	3.48E-02	1.87E-46	4.30E-02
**1/Area (1/um^2)**	2.27E-03	2.61E-04	4.98E-04	2.85E-04
**1/Unit Cost (1/$)**	1.6E+04	3.7E+02	7.0E+02	4.0E+02

Equations for Table S4:

$$Capacity = Bandwidth \times log_2(1 + SNR)$$

$$SNR = \frac{P_{signal}}{P_{noise}} = \frac{I_{min}}{Responsivity \times \sigma^2}$$

$$BER = P(0) \times P(1|light\ off) + P(1) \times P(0|light\ on) = 1 - \Phi(0.5\mu/\sigma) = 10^{-12}$$

$$P2P\ Latency = \sum device\ latency + light\ propagation\ latency$$

$$Energy = Laser\ energy + \sum device\ driving\ energy$$

3.4 Fundamental physical limits of CLEAR factors

Table S5 Link level physical limits

Factor	Capacity	P2P Freq.	Energy Eff.	Are Eff.	Cost Eff.
**Unit**	Gbps	THz	bit/fJ	$1/um^2$	1/$
**Physical Limit**	1.27E+16	3e-2 to 3	174216.03	2.83E+05	1.00E+10
**Physical law**	Bremermann's limit	Speed of light in Silicon	Landauer limit	Two neighboring atoms	Total human wealth
**Equation**	c^2/h	length/c	kT·ln2	$1/2\pi r^2$	Unbounded

The parameters in Table S5 are:

- Vacuum light speed $c = 3 \times 10^8$ m/s
- Refractive index of waveguide *n*

4. Compute Unit Model Analysis

4.1 Device level CLEAR model

The five-component device-level CLEAR FOM is comprised as follows:

1) **Capability:** The operating frequency of a device in the unit of [GHz] can be regarded as the capability of a device.
2) **Latency:** We replaced the latency with critical length, L, of the device, in the unit of [nm]. This is because the latency for each individual device is small, however, the critical length, which represents the length of the functional part of the device (e.g. gate length for transistor, perimeter of the photonic ring modulator and the modulation length of the plasmonic and HyPPI EOM), is also related to the latency due to the group index of the optical mode.
3) **Energy:** Energy consumption of a device in the unit of [fJ/bit] can be calculated by $\frac{1}{2}CV^2$ where C and V are the capacitance and the driving voltage of the device respectively.
4) **Amount:** Since all the devices on-chip are arranged on the same plane, the amount of a device only needs to consider its 2D area in the units of [um^2].
5) **Resistance:** The economic resistance on device level is the cost to fabricate such device in [$]. For transistors, the historical fabrication cost is known. However, for optical devices, there is no standardized fabrication cost readably available, since even Silicon photonics foundries cannot rely on high-volume data to date. Therefore, we estimated the optical device fabrication cost based on the total cost of an optical wafer (~$50,000) and the number of the device that can fabricate on a single chip (~70,000 mm^2).

4.2 Device Parameters

Table S6. Device level CLEAR data

	Electrical Transistor	Ring EOM	Plasmonic EOM	Hybrid EOM
**Freq. (GHz)**	1.1E+03	2.5E+01	5.9E+01	2.1E+03
**Scaling (1/um)**	3.3E+01	3.2E-02	5.0E-01	5.0E-01
**Energy (bit/fJ)**	1.8E+02	3.6E-01	1.5E-01	2.4E-01
**Area ($1/um^2$)**	4.4E+03	1.4E-02	2.5E-01	2.0E+00
**Cost (1/$)**	1.0E+09	8.3E+03	1.5E+05	1.2E+06

All the device data are taken from our previous work [R3].

4.3 Fundamental physical limits of CLEAR factors

Table S2 Device level physical limits

**Factor**	**Operating Frequency**	**Scaling Eff.**	**Energy Eff.**	**Are Eff.**	**Cost Eff.**
**Unit**	GHz	1/um	bit/fJ	$1/um^2$	1/$
**Physical Limit**	1.7E+04	6.7E+02	3.5E+05	5.7E+05	1.0E+10
**Physical law**	Margolus–Levitin theorem	Heisenberg Uncertainty Principle	Landauer limit	Single atom	Human wealth
**Equation**	h/4E	$\sigma_x\sigma_p \geq \hbar/2$	kT·ln2	$1/\pi r^2$	Unlimited

The parameters in Table S2 are:

- Planck constant $h = 6.626 \times 10^{-34}\ J \cdot s$
- Average energy of the system E
- Standard deviation of position σ_x
- Standard deviation of momentum σ_p
- Reduced Planck constant $\hbar = 1.055 \times 10^{-34}\ J \cdot s$
- Boltzmann constant $k = 1.381 \times 10^{-23}\ J \cdot K^{-1}$
- Temperature T in kelvins
- Radius of the atom r in micrometers

5. References

[R1] Data after http://www.singularity.com/charts/page62.html, accessed (3/6/2020).

[R2] Miller, D. A. (2017). Attojoule optoelectronics for low-energy information processing and communications. Journal of Lightwave Technology, 35(3), 346-396.

[R3] Sun, S., Badawy, A. H. A., Narayana, V., El-Ghazawi, T., & Sorger, V. J. (2015). The case for hybrid photonic plasmonic interconnects (HyPPIs): Low-latency energy-and-area-efficient on-chip interconnects. IEEE Photonics Journal, 7(6), 1-14.
